# The Medical Student; or, Aids to the Study of Medicine: Including a Glossary of the Terms of the Science, and of the Mode of Prescribing, Bibliographical Notices of Medical Works, the Regulations of the Different Medical Colleges of the Union, &c.

**Published:** 1837-10

**Authors:** 


ART. VII.—*The Medical Student; or, Aids to the Study of Medicine: including a Glossary of the Terms of the Science, and of the Mode of Prescribing, Bibliographical Notices of Medical Works, the Regulations of the different Medical Colleges of the Union, &c. &c.* By R. DUNGLISON, M.D. &c.—*Philadelphia*, 1837. 8vo. pp. 323.

THIS is another of those valuable compilations for which the profession in America is so much indebted to Professor Dunglison. Although chiefly intended for students in the American States, it will be useful to students in all countries, as it contains a vast deal of that kind of miscellaneous and varied information which is so constantly needed, yet so difficultly found by them. Besides the mere technical matters, this volume touches on many subjects of yet higher importance, and, among others, on the moral duties and professional conduct of the medical practitioner, which are laid down clearly and forcibly, and with a just appreciation of the dignity of the office. The following titles of the five chapters of which the work consists will give a general but not very exact notion of its contents, as it comprehends many things very interesting to the student, yet hardly suggested by its title:—Preliminary Education; Medical Education prior to attendance on Lectures; Medical Education during the period of attendance on Lectures; Medical Education after Graduation; A Medical Bibliography for the Student and young Practitioner.

We recommend "The Medical Student" in the strongest terms to his brethren in all countries, and in an especial manner to his compatriots.

